# Physiological Analysis and Transcriptome Sequencing Reveal the Effects of Salt Stress on Banana (*Musa acuminata* cv. BD) Leaf

**DOI:** 10.3389/fpls.2022.822838

**Published:** 2022-04-12

**Authors:** Junya Wei, Debing Liu, Yuewei Liu, Shouxing Wei

**Affiliations:** ^1^Tropical Crops Genetic Resources Institute, Chinese Academy of Tropical Agricultural Sciences, Haikou, China; ^2^Applied Science and Technology College, Hainan University, Haikou, China

**Keywords:** salt stress, banana, physiological analysis, transcriptomic analysis, effects

## Abstract

The salinization of soil is a widespread environmental problem. Banana (*Musa acuminata* L.) is a salt-sensitive plant whose growth, development, and production are constrained by salt stresses. However, the tolerance mechanism of this salt-sensitive banana to salt stress is still unclear. This study aimed to investigate the influence of NaCl treatment on phenotypic, physiological, and transcriptome changes in bananas. We found that the content of root activity, MDA, Pro, soluble sugar, soluble protein, and antioxidant enzymes activity in salt-stress treatment were significantly higher than the control in bananas. Transcriptome sequencing result identified an overall of 3,378 differentially expressed genes (DEGs) in banana leaves, and the Kyoto Encyclopedia of Genes and Genomes analysis indicated that these DEGs were involved in phenylpropanoid biosynthesis process, ribosome process, starch and sucrose metabolism, amino sugar process, and plant hormone signal transduction process that had simultaneously changed their expression under salt stress, which indicated these DEGs may play a role in promoting BD banana growth under salt treatments. The genes which were enriched in the phenylpropanoid biosynthesis process, starch and sucrose metabolism process, amino sugar process, and plant hormone signal transduction process were specifically regulated to respond to the salt stress treatments. Here, totally 48 differentially expressed transcription factors (TFs), including WRKY, MYB, NAC, and bHLH, were annotated in BD banana under salt stress. In the phenylpropane biosynthesis pathway, all transcripts encoding key enzymes were found to be significantly up-regulated, indicating that the genes in these pathways may play a significant function in the response of BD banana to salt stress. In conclusion, this study provides new insights into the mechanism of banana tolerance to salt stress, which provides a potential application for the genetic improvement of banana with salt tolerance.

## Introduction

In the process of plant growth, plants will inevitably face various biotic and abiotic stresses. In general, abiotic stress reduced crop yield by more than 50%, while biological stress reduced crop yield by less than 10% (Kreps et al., 2002). Salt is one of the most significant abiotic factors, which is a serious threat to crop yield worldwide, and it limits the growth and productivity of crops. Soil salinization is one of the most common abiotic stresses in agricultural production worldwide. Due to climate change and limited rainfall, the degraded area with saline soils has rapidly increased, which has led to great challenges facing global food security (Godfray et al., 2010). In recent years, the area of land salinization has become a worldwide problem, which poses a serious threat to the ecological environment and agricultural production. Currently, about 950 million hectares of cultivable land in the world is suffering due to salt, and more than 11% of irrigated areas of cultivable land have been salinized due to irrigation (Abdel Latef and He, 2011; Wang et al., 2017). Salt preserves high tension soil water and nutrients, which adversely impacts plant growth and development. To adapt to the changes in the living environment, plants will adjust to physiological and biochemical changes and gene expression accordingly. To cope with the salt stress and survive it, plants have adopted a series of complex strategies, which include outflow of ions, improvement of antioxidant enzyme activity, and the synthesis of osmotic regulatory proteins (Lv et al., 2019). Therefore, understanding the regulation mechanism of salt stress response and identifying salt resistance genes in plants are very necessary for using genetic engineering technology to improve salt tolerance in crop plants.

Transcriptome analysis is a tool used to quantify the mRNA expression standard on account of next-generation DNA sequencing technology, which can distinguish the key genes involved in various kinds of biological processes (Fan et al., 2018). RNA sequencing technology with high-throughput sequencing has been applied in plants to study the response genes or key pathways of these plants under abiotic or biological stress (Chen et al., 2014). In recent years, with the maturity of high-throughput sequencing technology, transcriptome research has been carried out in many plants, and made remarkable progress has been made in revealing the mechanism of plant stress response and mining of key genes. Transcriptome analysis based on high-throughput sequencing can reveal the molecular mechanism of plant salt tolerance. Transcriptome analysis of Canola (*Brassica napus*) roots under saline conditions state clearly that the number of differentially expressed genes (DEGs) under salt stress was 163 (Long et al., 2015). Transcriptome analysis of two contrasting rice cultivars during alkaline stress displayed that a total of 9,078 unique DEGs were identified, which were divided into 15 disjointed subgroups (Li et al., 2018). Transcriptional analysis of salt-tolerant common soybean under saline conditions showed that 6,422 and 4,555 unigenes were differentially expressed in leaf and root tissues, respectively (Hiz et al., 2014). Transcriptome analysis has been widely used in eukaryotes, such as Arabidopsis (Lister et al., 2008), *Caenorhabditis elegans* (Hillier et al., 2009), rice (Zhang et al., 2010), and *Vitis vinifera* (Zenoni et al., 2010).

Banana (*Musa acuminata* L.) belongs to perennial monocotyledonous herbaceous plant of the order *Zingiberales*, which is not only the most widely consumed fruit but also the staple food of millions in many tropical countries and subtropical countries worldwide, which is vital for food security around the world. Like other crops, banana is adversely affected by pathogens and suboptimal cultivation environments. Banana plant has a relatively fast growth rate, everlasting green canopy, and shallower roots which are very sensitive to water stress caused by salt stressors compared with some other crops. Salt stress will lead to slow growth and this causes significant yield loss in bananas. The physiological response to salt stress is under control with a large deal of genes expression, and the study of genetic regulation concerning salt-induced stress responses can help us to manage banana plants and improve soil salinity caused by irrigation and climate change. Some genes in response to salt stress of banana plants had been identified at the transcriptional level previously. Lee et al. (2015) found that the expression of target proteins was related to various biological processes, such as stress signaling, stress defense, transportation, intracellular homeostasis, metabolism, and other stress-related functions, that increased with salt stress based on RNA-seq. BD banana (*Musa acuminate* L. AAA group cv. Cavendish) is a banana variety selected by the Chinese Academy of Tropical Agricultural Sciences from the offspring plants of tissue culture for many years. It is widely cultivated in the tropical and subtropical regions of China and they have a high resistance to *Fusarium Wilt* and the advantages of high and stable yield (Zhang et al., 2014). As the plant is very sensitive to salt stress, the yield of bananas reduced by half and the plant height decreased by about 75% under salt stress conditions (Israeli et al., 1986; Yano-Melo et al., 2003). The purpose of this study was to study the salt tolerance mechanism of banana plants through the physiological and transcriptome analysis of BD banana.

## Materials and Methods

### Plant Material and Salt Stress Treatments

Young BD plants propagated by tissue culture, unfolded at the five-leaf state, and with no diseases and pests used in this study were obtained from a banana tissue culture center (Institute of Banana and Plantain, Chinese Academy of Tropical Agricultural Sciences, DZ). The culture soil was removed and the roots were washed. Phenotypically uniform banana seedlings were transplanted into plastic containers with thermally sterilized vermiculite and half-strength Hoagland growth nutrient solution in the greenhouse (16 h light/8 h dark cycle; 200 μmolm^–2^s^–1^ light intensity; 28°C; and 70% relative humidity). The plants were irrigated twice daily with half-strength Hoagland nutrient solution to maintain constant nutrient concentrations. One week after growth in the greenhouse, the banana seedlings were treated with a half-strength Hoagland growth nutrient solution added with 60 mmol/L NaCl or no NaCl as the control. The BD banana seedlings were irrigated with proper nutrient solution two to three times a day until the solution was discharged freely from the bottom of the container. After washing with deionized distilled water, the materials of each treatment were gathered in the mine time of three independent control plants and salt-stressed plants at distinct time intervals like 5, 10, 15, 20, and 25 days to generate the final samples for relevant physiological analysis. Leaves samples were harvested from both salt-treated and control plants 10 days after treatments, frozen with liquid nitrogen, and stored at –80°C in the refrigerator immediately prior to cDNA library preparation and RNA-seq analyses. Three biological replicates were used in each treatment.

### Physiological Analyses

The relative water content (RWC), chlorophyll content, leaf membrane permeability, root activity, free proline content, soluble sugar content, soluble protein content, SOD activity, POD activity, and malondialdehyde content of the leaves were measured, respectively. For relative water content (RWC) determination, the fresh banana leaves were measured as fresh weight (FW). Then the BD banana leaves were dried to a constant weight at 75°C for 72 h and the leaves were weighed as dry weight (DW). Relative water content was calculated using the equation: RWC (%) = (FW—DW)/FW × 100. The permeability of leaf plasma membrane was measured by conductometer method (Gao, 2006), the content of soluble sugar was measured by anthrone colorimetry (Gao, 2006), the proline content was measured with acid–ninhydrin method (Gao, 2006), and the content of malondialdehyde (MDA) was measured by TBA colorimetry (Gao, 2006). The root activity was measured by TTC (triphenyltetrazolium chloride), the proline content was measured by acid ninhydrin method, the soluble sugar content was measured by anthrone-colorimetry method, the soluble protein content was measured by Coomassie brilliant blue method, the MDA content was measured by thiobarbituric acid colorimetry, the SOD activity was measured by nitro blue tetrazolium chloride (NBT) reduction method, the POD activity was measured by guaiacol method. In each analysis, there were three replicates, and three seedlings were combined together as one replicate.

### RNA Extraction, Library Construction, and RNA-Seq

Firstly, the salt-stress treated samples and control samples were mixed, respectively, and used for RNA preparation. Total RNAs from BD banana leaves sample were isolated using RNeasy Plant kit (Qiagen, Hilden, Germany). The RNA samples’ concentration was determined with NanoDrop 1,000 spectrophotometer at A_260/280_ (Thermo Fisher Scientific, Wilmington, DE, United States), meanwhile RNA quality was assessed by 1% denatured agarose gel electrophoresis. The RNA integrity was assessed according to the RNA Nano 6000 Assay Kit of the Agilent Bioanalyzer 2100 system (Agilent Technologies, CA, United States). RNA-seq libraries were produced using Illumina HiSeq™ 2000 sequencing performed by the Biomarker Biotechnology Corporation (Beijing, China).

### Quality Control, Comparative Analysis, and Gene Function Annotation

Raw data of fastq format were firstly processed through in-house perl scripts. In this step, clean data were obtained by removing reads containing adapter, reads containing ploy-N, and low-quality reads from raw data. At the same time, Q20, Q30, GC-content, and sequence duplication levels of the clean data were calculated. All the downstream analyses were based on clean data with high quality. The adaptor sequences and low-quality sequence reads were removed from the data sets. Raw sequences were transformed into clean reads after data processing. These clean reads were then mapped to the reference genome sequence (DH_Pahang_v2) using HISAT2 software. Gene function was annotated based on the following databases: Nr, Nt, KOG/Clusters of Orthologous Groups (COG), Swiss-Prot, Kyoto Encyclopedia of Genes and Genomes (KEGG), and Gene Ontology (GO).

### Identification of Differentially Expressed Genes and Functional Enrichment Analysis

Differential expression analysis was carried out using the DESeq2 (Anders and Huber, 2010; Love et al., 2014) to provide statistical routines to determine the differential expression. The method of Benjamin and Hochberg is used to control the error detection rateto adjust the generated *p*-value. DEGs were genes with adjusted *p* < 0.01 found by DESeq2. Fragments per kilobase of exon per million fragments mapped (FPKM method) were used to calculate the gene expression level (Trapnell et al., 2010). The DEGs between no NaCl or 60 mmol/L NaCl samples were identified, and gene abundance differences were calculated with the ratio of the FPKM values. The false discovery rate (FDR) control method was used to identify the threshold of the *p*-value to calculate the significance of the differences. Differential expression analysis of the two samples was performed using the edgeR. The FDR < 0.01 and fold change ≥ 2 was set as the threshold for significant differential expression. The DEGs were searched with the GO database, the COG database, and the KEGG database. Then, GO enrichment analysis of the DEGs was implemented by the GO seq R packages based Wallenius non-central hyper-geometric distribution (Young et al., 2010), which can adjust for gene length bias in DEGs. Furthermore, the KOBAS (Mao et al., 2005) software was used to test the statistical enrichment of DEGs in KEGG pathways.

### Real-Time Quantitative PCR Analysis

To confirm the RNA-seq results, real-time quantitative PCR (RT-qPCR) was carried out using LightCycler^®^ 96 real-time PCR system (Roche, Basel, Switzerland). The primers were designed and listed in Table 1. The UBQ2 (GenBank accession no. XP009390884.1) gene was selected as the internal control (Chen et al., 2011). The relative expression levels of the target genes were calculated by the 2^–ΔΔCt^ method (Livak and Schmittgen, 2001).

**TABLE 1:** Primers used for qRT-PCR analyses.

Gene ID	Upstream primer	Downstream primer
Ma10_g29990	GCGGCGGCAGCAGCAGTAGCAGTGG	GCGGCGGCAGCAGCAGCAGCAGTGG
Ma05_g23640	CATGGAGATCATGGAGGCC	CATGGAGATCATGGAGGCC
Ma06_g06920	GAGACCCCAAAGGAGGAGAAAG	GAGACCCCAAAGGAGGAGAAAG
Ma03_g27360	GTGGTGAAGAAGCTGAACT	GTGGTGAAGAAGCTGAACT
Ma09_g04890	CTTCCACCCCACCGACGAGG	CTTCCACCCCACCGACGAGG
Ma07_g20020	GATGGAAGAACAGGAGAAGC	GATGGAAGAACAGGAGAAGC
Ma02_g20220	TCCAGCAGCTGCTGCAGCG	TCCAGCAGCTGCTGCAGCG
Ma06_g32210	GGAGGCGGAGGAGGAGGGGC	GGAGGCGGAGGAGGAGGGGC
Ma05_g08940	TCCTCTTCCCCATCATCATCATCATCTTCT	TCCTCTTCCTCTTCATCATCATCATCTTCT
Ma11_g16180	GCCAACGCCAACGCCAACGCCG	GCCAACGCCAACGCCAACGCCG
MaUBQ2	GGCACCACAAACAACACAGG	AGACGAGCAAGGCTTCCATT

## Results

### Performance Response of BD Banana Under Salt Treatments

**FIGURE 1:**
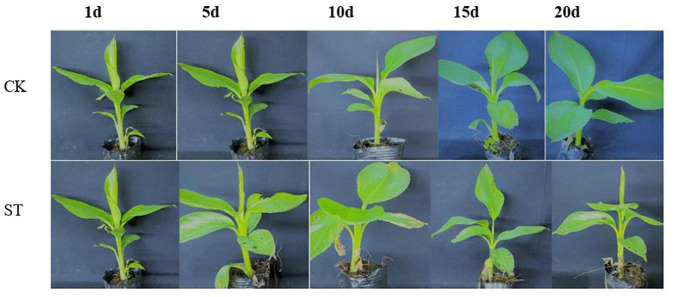
Effect of NaCl treatment on BD banana seedling growth. Five leaf stage banana seedlings were subjected to 60 mM NaCl treatments and photos were taken at different time points.

To accurately evaluate the tolerance of salt stresses of BD, young banana seedlings of BD at the five-leaf state with uniform growth were treated with salt stresses. On treatment with 60 mM NaCl, the leaves of BD banana turned brown with chlorosis and necrosis of the edges, and with the extension of salt stress time they gradually withered. However, no obvious damage was observed in BD banana leaves under 0 mM NaCl treatment. Banana exposed to the salt stresses for 20 days showed slower growth than the control (Figure 1).

### Physiological Characteristics of BD Banana Under Salt Treatments

**FIGURE 2:**
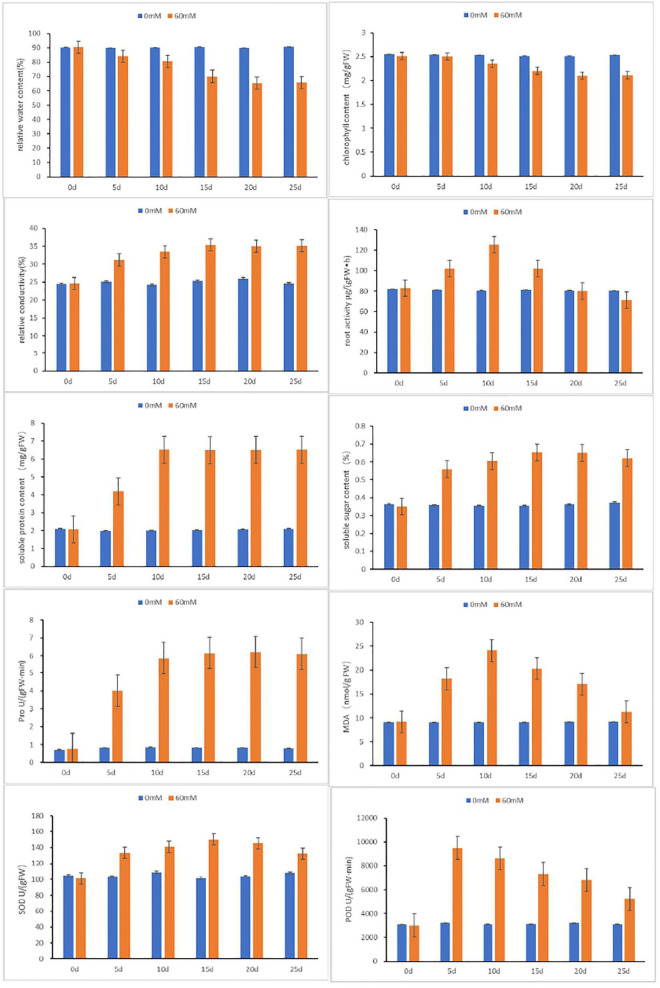
Physiological analyses of BD in response to salt stresses at 0, 5, 10, 15, and 20 days after treatments. Relative water content, chlorophyll content, relative conductivity, root activity, Pro, soluble sugar content, soluble protein content, MDA, S OD and POD were examined under normal and treated conditions. Data are means ± SD calculated from three independent experiments.

The physiological and biochemical functions of plants are significantly affected by salt stress. To further accurately evaluate physiological response of BD banana under salt treatment, several physiological indices were measured (Figure 2). By contrast, both the relative water content and chlorophyll content displayed notable decline when treated with 60 mM NaCl. The downtrend became slow after 10-day treatment, which suggested that salt stress led to the injury of banana leaves. It was indicated that relative conductivity, soluble sugar content, and soluble protein content fluctuate with a slow rise. The root activity and MDA content were even higher in salt-treated BD banana plant than in control plant, and has a maximal value at 10 days, while the content of Pro rose sharply and leveled off after 10 days. The two oxidases detected in this study were significantly different between salt-treated and control BD banana plant. The activities of SOD and POD in salt treatment BD banana plant were always higher than the control. Acting as an antioxidant system enzyme, POD plays an important role in the function of hydrogen peroxide detoxification and lignin biosynthesis (Chibbar and Vanhuystee, 1984; Gao et al., 2010). POD activity increased significantly under salt stress (Jain and Bhatla, 2018). This result is consistent with ours. The activities of SOD and POD in the leaves of salt-treated BD banana plant increased sharply from 0 to 5 days, and the activity of POD increased sharply from 0 to 5 days, then decreased after 5 days when compared to the untreated control. The fluctuation of root activity, MDA, Pro content, and antioxidant enzymes activity in salt-treated BD banana plant indicate that a material change in salt-sensitive gene expression will happen 10 days after treatment with 60 mM NaCl. Therefore, RNA-seq analysis was performed on these leaf samples (i.e., 0 and 10 days).

### *De novo* Assembly of the Sequencing Data

**FIGURE 3:**
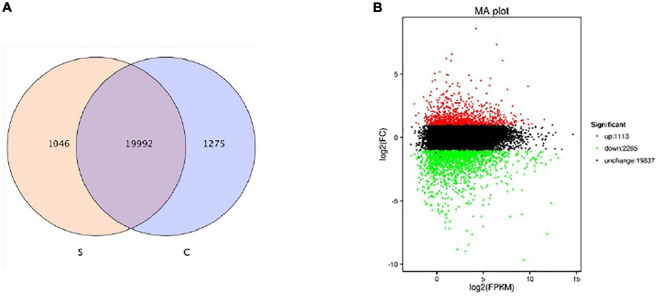
Survey of expressed genes **(A)** and gene expression level **(B)** of BD banana between the salt stress-treated with 60 mM of NaCl and control libraries. Reel dots represent transcripts more prevalent in the library of salt-treated one, green dots show those present at a lower frequency in salt-treated plant and black dots indicate transcripts that did not change significantly.

To further research the molecular mechanisms related to the physiological response of BD banana in salt-stress state, we constructed six cDNA libraries with no NaCl-treated BD banana plant and 60 mM NaCl-treated BD banana plant on the 10th day and sequenced using the Illumina sequencing platform. Our previous data demonstrated that the detectable physiological effects of salt stress became significant on the 10th day of treatment in hydroponic culture when treated with 60 mM NaCl of BD banana plant (Figures 1, 2). Therefore, leaf RNA samples at this time point were collected and used for RNA-seq analysis. We performed Illumina sequencing for each RNA sample (C1, C2, C3, S1, S2, and S3) and generated six subtranscriptomes. The raw data of fastq format were firstly processed through in-house Perl scripts from sequencing. At the same time, Q20, Q30, GC-content, and sequence duplication levels of the clean data were calculated. Then the adaptor sequences and low-quality sequence reads were removed. A total of 180 million clean reads were obtained from the six subtranscriptomes (Table 2) with the Q20 percentage for each data being 98.38, 98.2, 98.1, 98.39, 98.24, and 98.1%, respectively and the GC ratio (%) for each library was 52.31, 52.33, 52.06, 50.72, 51.28, and 51.16% that constituted over 53 GBase (27.17034 nt in BD banana plant control sample, and 26.66391 nt in salt treatment BD banana plant sample) of data. After quality evaluation and data filtering, the data provided rich information for the subsequent analysis of salt stress-related genes in BD banana plant. As a result, a total of 22,313 genes were obtained between control BD plants (untreated) and salt stress-treated BD plants. Among these expressed genes, there were 19,992 genes that were expressed in both the samples. In addition, 1,046 and 1,275 genes were only present in salt-treated BD banana plant and control BD banana plant, respectively (Figure 3A). Among these genes uniquely expressed in salt stress BD banana plant, some important genes were observed to participate in abiotic stress response for such transcription factors (TF).

**TABLE 2:** Statistics of raw data for BD banana transcriptome.

Samples	Total reads	Total nucleotides (nt)	Q20 percentage	GC percentage
C1	28,949,011	8,639,530,664	98.38	52.31
C2	32,460,424	9,684,397,870	98.2	52.33
C3	29,663,510	8,846,412,260	98.1	52.06
S1	29,276,283	8 680 826 602	98.39	50.72
S2	30,792,897	9,166,374,030	98.24	51.28
S3	29,546,663	8,816,704,996	98.1	51.16

### Annotation of Transcript Sequences by KOG and Gene Ontology

**FIGURE 4:**
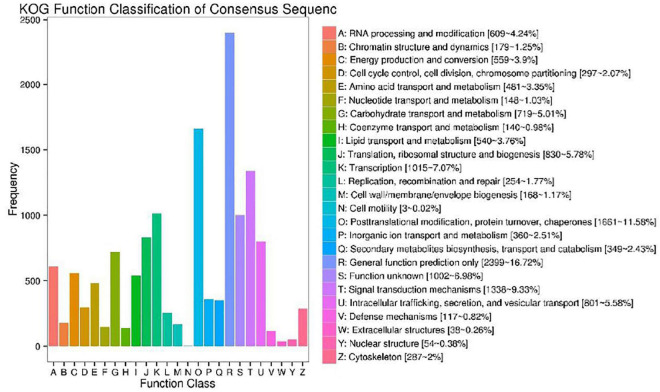
Cluster of orthologous groups for enkaryotic complete genomes (KOG) classification. A total of 14,348 sequences with KOG classifications within the 25 categories are shown.

**FIGURE 5:**
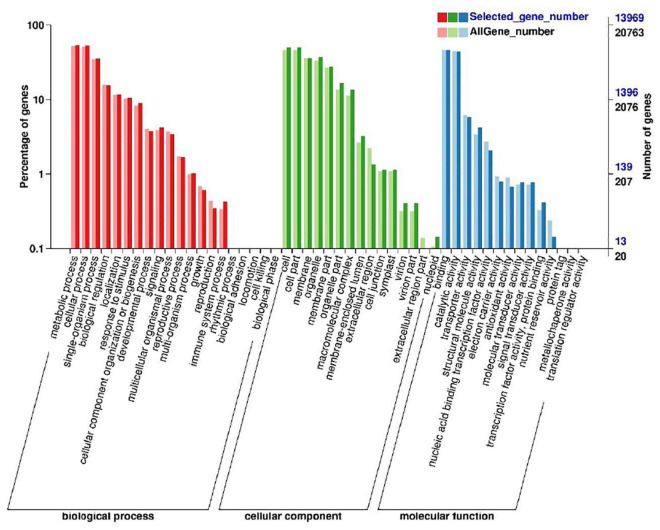
BD banana functional classification by gene ontology (GO) analysis. A total of 13,969 unigenes have been assigned GO terms and they are classified into three GO categories: biological process, cellular component, and molecular function.

We used the KOG database to classify the functions of transcriptional sequences. A total of 14,348 single genes were annotated into 25 precise KOG categories, as shown in Figure 4. The first three categories were “General function prediction only” with 2,399 single genes (16.72%), “Posttranslational modification, protein turnover, chaperones” with only 1,661 single genes (11.58%), and “Signal transduction mechanisms” with 1,338 single gene (9.33%). The correlative KOG categories which responded to salt stress are the follows: “Defense mechanisms” (0.82%), “secondary metabolites biosynthesis, transport and catabolism” (2.43%), “inorganic ion transport and metabolism” (2.51%), “lipid transport and metabolism”(3.76%), “coenzyme transport and metabolism” (0.81%), “carbohydrate transport and metabolism”(0.98%), “nucleotide structure”(0.38%), and “amino acid transport and metabolism” were 3.35%. We also used the GO analysis to classify the functions of transcriptional sequences of 13,969 single genes (Figure 5). Among them, 37.24% belong to the “biological process category,” 43.46% belong to the “cellular component category,” and 19.30% belong to the “molecular function category.” In the comparison of GO classification, the main classifications of biological processes are “metabolic process,” “cellular process,” “single-organism process,” “biological regulation,” and “localization.” The main classifications of cellular components are “cell,” “cell part,” “membrane,” and “organelle.” The main classifications of molecular function are “binding,” “catalytic activity,” “transporter activity,” “structural molecule activity,” and “nucleic acid binding transcription factor activity.” In addition, a total of 5,324 (26.63%) single genes were grouped into 144 biochemical pathways through the KEGG database, and these single genes were mainly located in “ribosome” (ko03010, 459 single genes), “Carbon metabolism” (ko01200, 301 single genes), “Plant hormone signal transduction” (ko04075, 295 single genes), and “Biosynthesis of amino acids” (ko01230, 279 single genes). The other significant pathways bound with abiotic stress response are as follows: “Arginine and proline metabolism” (ko00330), “cysteine and methionine metabolism” (ko00270), “glycine, serine and threonine metabolism” (ko00260), “phenylpropane biosynthesis” (ko00940), etc.

### Identification of Differentially Expressed Genes Under Salt Stress in BD Banana

Salinity has a wide impact on the activity of plant cells, resulting in water deficit and ion stress, including inhibition of essential enzymes, instability of cell membrane, reduction of nutrient supply, etc. (Zhu, 2002; Hasegawa and Bressan, 2011). We found and identified a large number of differentially expressed transcripts by comparing six subtranscriptomes. We also made the differential expression analysis based on the expression abundance value, and 3,378 DEGs with significantly different expression levels between 60 mM NaCl treatment samples and control samples of BD banana plant (*Musa acuminata* L.) were detected in the end. There were 1,113 up-regulated DEGs and 2,265 down-regulated DEGs among 60 mM NaCl-treated BD banana plant samples and control samples of BD banana (Supplementary Table 1). The gene expression levels with 60 mM NaCl treatment samples and control samples of BD banana plant are shown in Figure 3B according to the value of expression abundance (RPKM).

### Gene Ontology Function Analysis of Differentially Expressed Genes

**FIGURE 6:**
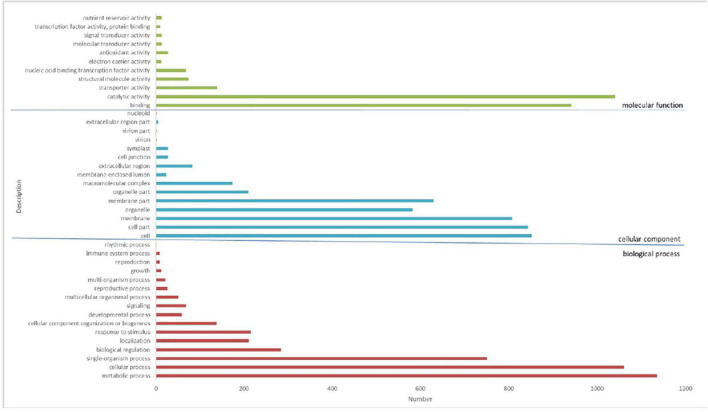
Gene ontology (GO) classification of DEGs by analysis. A total of 2,065 DEGs have been assigned GO terms and they are classified into three GO categories: biological process, cellular component, and molecular function.

**FIGURE 7:**
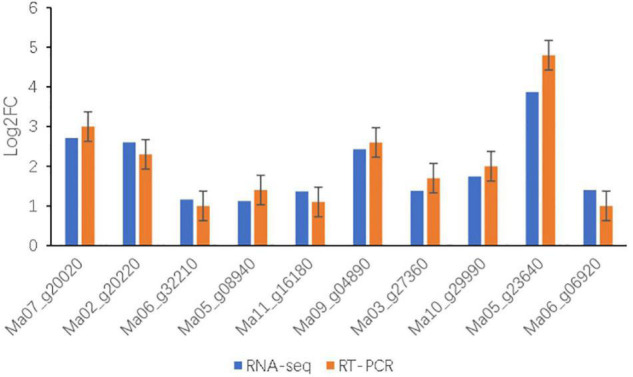
Relative expression levels of 10 genes after salt treatments in BD banana were examined by qRT-PCR. The relative gene expression levels were determined by 2^–ΔΔCT^. Transcript levels were normalized to the expression level of UBC. Data are means ± SD of *n* = 3 independent experiments.

As a common functional classification system, GO can be used to determine the main molecular functions related to the DEGs genes. Ultimately, 2,065 of the 3,378 DEGs were divided into 42 groups of three major categories, biological process, cellular component, and molecular function (Figure 6). In the category of biological process, most DEGs genes are divided into the metabolic process (approximately 33.6%) and cellular process (approximately 31.4%), then by single biological process (approximately 22.2%), biological regulation (approximately 7.6%), stimulus-response (approximately 6.4%), and localization (approximately 6.3%). In the molecular functions category, 30.8% of the DEGs genes were assigned to catalytic activity, followed by binding (approximately 27.9%) and transporter activity (approximately 0.4%). In terms of cell component categories, cell (approximately 25.2%) was the major group, then by cell part (approximately 24.9%), membrane (approximately 23.9%), membrane part (approximately 18.6%), organelle part (approximately 6.2%) (Figure 7). The transcriptional changes of metabolic and cellular processes in certain environmental conditions indicated that a wide range of metabolic activities will occur when the plant suffered under stress conditions (Bowman et al., 2013; Mousavi et al., 2014).

### Enrichment Analyses of Differentially Expressed Genes

We used the GO database to classify the enrichment analysis of these DEGs in BD banana transcripts to understand the function of these DEGs and enriches of 72 GO items (*P* ≤ 0.01), an integral component of membrane (5,135 DEGs), followed by anion binding (2,778 DEGs), carbohydrate derivative binding (2,366 DEGs), carbohydrate metabolic process (1,084 DEGs), and transferase activity, transferring glycosyl groups (548 DEGs). The other significantly enriched GO terms concerned with stress response were as follows: hydrolase activity, hydrolyzing O-glycosyl compounds, transferase activity, transferring glycosyl groups, extracellular region, and coenzyme binding. This result suggested that the GO terms containing DEGs might occupy an important position in the molecular mechanism of salt resistance of BD banana plant.

### Kyoto Encyclopedia of Genes and Genomes Pathway Analysis of Differentially Expressed Genes

**FIGURE 8:**
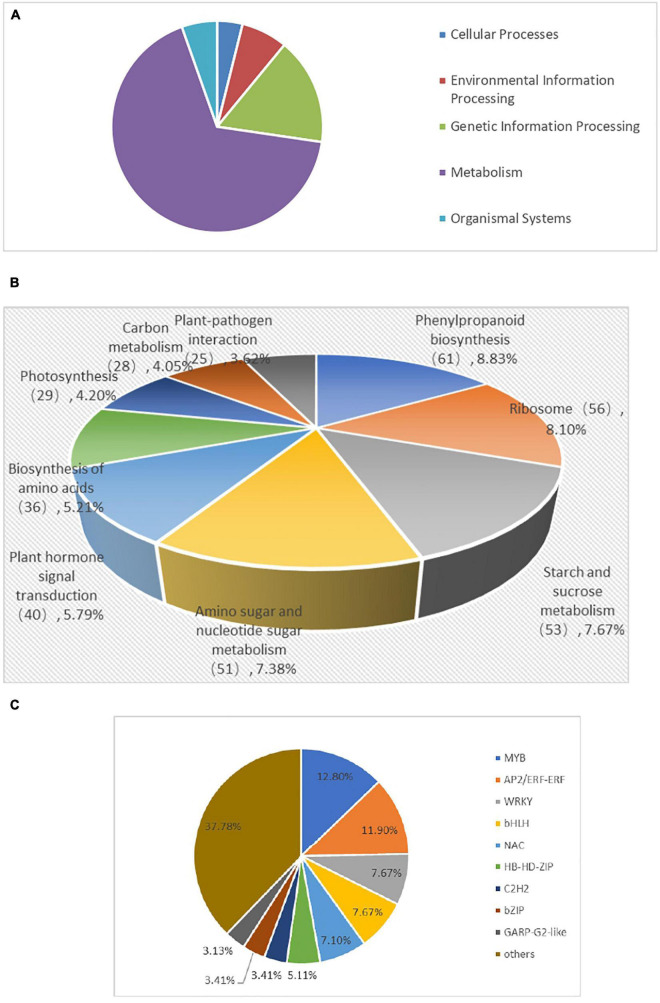
KEGG classification **(A)**, KEGG enriched pathways **(B)**, and Distribution of transcription factor gene families expressed **(C)** of DEGs inBD banana.

To comprehensively understand the lively biological pathways in DEGs between 60 mM NaCl-treated samples and control samples of BD banana plant (*Musa acuminata* L), the affected biochemical pathways were analyzed according to the expression profile. The main distinguished biological pathways of these DEGs in BD banana plant are displayed in Supplementary Table 2. The results showed that 691 DEGs out of the 3,378 DEGs were annotated to 112 metabolic pathways. Among which, 439 up-regulated DEGs genes were annotated into 90 metabolic pathways while 637 down-regulated DEGs genes were annotated into 92 metabolic pathways. There were 20 metabolic pathways annotated as up-regulated DEGs, 22 metabolic pathways annotated as down-regulated DEGs down, and 70 metabolic pathways annotated as both up-regulated and down-regulated genes at the same time among the 112 metabolic pathways. The 112 KEGG pathways were covered with the five main KEGG categories, including cellular processes, environmental information processing, genetic information processing, metabolism, and organismal systems (Supplementary Table 3 and Figure 8A). The DEGs were carried out additionally distinguished based on their different biological functions. These DEGs mainly belong to KEGG pathways, including phenylpropanoid biosynthesis, ribosome, starch and sucrose metabolism, amino sugar and nucleotide sugar metabolism, plant hormone signal transduction, biosynthesis of amino acids, photosynthesis, carbon metabolism, cysteine and methionine metabolism, and photosynthesis–antenna proteins, which are important pathways in response of plant to abiotic and biotic stress that are reported in previous studies (Goyal et al., 2016; Zhang et al., 2017). Among the significantly enriched pathway of DEGs, phenylpropanoid biosynthesis (61 members for BD banana) was the largest complex. It was followed by ribosome (56 members for BD banana), starch and sucrose metabolism (53 members for BD banana), amino sugar and nucleotide sugar metabolism (51 members for BD banana), plant hormone signal transduction (40 members for BD banana), and so on (Table 3 and Figure 8B). The phenylpropane pathway is a kind of metabolic pathway with the maximum number of genes that can synthesize different plant secondary metabolites and play a role in the process of development and stress response. The accumulation of flavonoids is a sign of the plant in adverse circumstances (Winkel-Shirley, 2002). In this study, there were 21 DEGs with KEGG annotation in the flavonoid biosynthesis pathway. Furthermore, fatty acid elongation, cutin, suberine and wax biosynthesis, phenylalanine metabolism, flavone and flavonol biosynthesis, and Cyanoamino acid metabolism were all annotated DEGs.

**TABLE 3:** KEGG pathways of DEGs.

#	ID	Pathway	Annotation (691)	*p*-value
1	ko00196	Photosynthesis—antenna proteins	20 (2.89%)	8.39E-13
2	ko00940	Phenylpropanoid biosynthesis	61 (8.83%)	7.95E-11
4	ko00195	Photosynthesis	29 (4.20%)	1.43E-07
5	ko00941	Flavonoid biosynthesis	21 (3.04%)	2.45E-07
6	ko00062	Fatty acid elongation	17 (2.46%)	3.10E-07
7	ko00520	Amino sugar and nucleotide sugar metabolism	51 (7.38%)	4.49E-07
8	ko00073	Cutin, suberine and wax biosynthesis	12 (1.74%)	3.58E-05
9	ko00360	Phenylalanine metabolism	15 (2.17%)	0.000141044
10	ko00500	Starch and sucrose metabolism	53 (7.67%)	0.000310514
11	ko00460	Cyanoamino acid metabolism	6 (0.87%)	0.007648299
12	ko00514	Other types of O-glycan biosynthesis	2 (0.29%)	0.078282551
13	ko00902	Monoterpenoid biosynthesis	3 (0.43%)	0.084880135
14	ko00944	Flavone and flavonol biosynthesis	7 (1.01%)	0.010441539
15	ko00945	Stilbenoid, diarylheptanoid, and gingerol biosynthesis	8 (1.16%)	0.010135819
16	ko00960	Tropane, piperidine, and pyridine alkaloid biosynthesis	4 (0.58%)	0.107953865
17	ko00905	Brassinosteroid biosynthesis	5 (0.72%)	0.07867997

### Differentially Expressed Genes Encoding Transcription Factors

Transcription factors (TFs) are involved in responses induced by biotic and abiotic stress tolerance of plants and regulate gene expression as an upstream regulator in the metabolic pathways (Shan et al., 2013). In this study, to evaluate the complex signal pathway network in responses induced by salt stress, we made further comparison in the expression profiles of the TFs of BD banana plant. Furthermore, 3,378 DEGs were annotated into 48 transcription factor families (Supplementary Table 4). The number of genes encoding putative TFs is 352, and they play vital functions in responses induced by salt stress response through regulating the transcription of downstream genes responsible for plant tolerance in BD banana plant. The TFs were separated into 20 groups according to homolog classification (Figure 8C). In addition, five TF families accounted for 47.16% of these groups, including MYB (45 numbers), AP2/ERF-ERF (42 numbers), WRKY (27 numbers), bHLH (27 numbers), and NAC (25 numbers), and played important roles in response to salt-stress tolerance in BD banana plant. It has been proved that MYB, AP2/ERF-ERF, WRKY, bHLH, NAC TFs take part in plant defense response which plays major functions in answer to biotic or abiotic stress responses (Alves et al., 2014; Nakashima et al., 2014). The MYB transcription factors family has a large number of proteins that are diverse functionally found in all the eukaryotes. Most of the MYB proteins have been shown to be involved in regulating different cellular processes and function as transcription factors in biotic and abiotic stress responses (Stracke et al., 2001). According to the results obtained, 45 MYB transcription factors are induced by the salt stress response, including 4 upregulated MYB transcription factors genes and downregulated 41 MYB genes in BD banana plant. NAC gene is a kind of plant-specific transcription factor, which contains a highly conserved N-terminal domain called the NAC domain. According to previous studies, some of these NAC genes have been defined as participating in salt stress response (Balazadeh et al., 2011; Mahmood et al., 2016). In this research, 25 NAC domain genes were found in DEG that are induced by salt stress response, including 11 upregulated NAC genes and 14 downregulated NAC genes, in BD banana plant. As a molecular chaperone, heat shock protein (HSP) plays a part in protecting plants against the biotic or abiotic stress atmosphere (Zhang et al., 2015). Here, we also found 15 HSPs in the differentially expressed genes with 12 up-regulated HSPs and 3 down-regulated HSPs. These transcription factor genes may contribute to the study of the molecular mechanism of salt resistance of BD banana plant.

### Validation of RNA-Seq Analysis by Quantitative RT-PCR

To further validate the reliability of sequencing data by transcriptome analysis under salt treatment of BD banana plant, 10 DEGs were randomly selected for qRT-PCR analysis (Figure 7 and Table 1). The results showed that the expression patterns of these DEGs obtained by qRT-PCR were consistent trends with the results of the RNA-seq. These results proved that the method for confirming DEGs of this experiment is reliable.

## Discussion

Adverse effects of salt stress on crops are nutrient limitations, ion toxicity, and oxidative and osmotic stresses (Shrivastava and Kumar, 2015). Salinity tolerance is under the control of multiple genes as a quantitative trait. Plants achieved salt tolerance through integrated physiological, cellular, molecular, and metabolic level responses. BD banana (*Musa acuminata* L. AAA genotype) is the main banana variety in China at present and has broad prospects for popularization and application (Zhang et al., 2014). The main environmental stress which affects the growth and productivity of banana plant is soil salinity. Understanding the molecular and physiological mechanisms of salt stress response tolerance is advantageous to promote the growth of banana plants under salt-stress conditions. The leaves of BD banana turned brown with chlorosis and necrosis of the edges as leaves symptoms and with the extension of salt stress time they gradually wilt than the control (Figure 1). The determination of the physiological response states clearly that the content of root activity, MDA, Pro, soluble sugar, soluble protein, and antioxidant enzymes activity in salt stress treatment were significantly higher than the control. Transcriptome analysis is a useful tool to distinguish salt stress response genes because it can comprehensively analyze gene transcription. We can better apply new agronomic and breeding practices to improve their adaptability to stress response after expounding the mechanism in biotic or abiotic stress conditions (Zhao et al., 2018). A comprehensive study of the transcriptional response of BD banana plant in salt stress treatment is helpful to clarify the regulatory network of salt stress adaptation and obtain candidate genes for increasing salt stress tolerance of the banana plant. Therefore, we assessed the transcriptome sequences between 60 mM NaCl-treated samples and control samples of BD banana plant (*Musa acuminata* L.), explored the variation of DEGs and biological metabolic pathways, and also looked for the candidate genes related to salt stress resistance. Our previous data demonstrate that the detectable physiological effects of salt stress became significant on the 10th day of treatment in hydroponic culture when treated with 60 mM NaCl of BD banana plant (Figures 1, 2). Therefore, leaf RNA samples at this time point were collected and used for RNA-seq analysis. In this study, we identified 1,046 expressed tolerance genes in BD banana plant after salt treatments (Figure 3). This study mainly studied the gene expression in the leaves of BD banana plant, and the selection of materials has certain limitations. There were differences in the expression of DEGs between the treatment sample and the control sample. The most plentiful DEGs were related to the metabolic process in the salt treatment of BD banana plant. The results of GO enrichment analysis help us to realize the function of these DEGs.

The hormone signal transduction pathway included zeatin biosynthesis, tryptophan metabolism, diterpenoid biosynthesis, and carotenoid biosynthesis which is a KEGG pathway directly involved with plant metabolism (Yan et al., 2020). In plant photosynthesis, carotenoids have two important functions: one is to participate in the light absorption of precursor cells, and the other is to prevent the light oxidation of precursor cells (Muller et al., 2001). Meanwhile, carotenoids can respond to the outside stimulus as signaling molecule precursors of plants (McNulty et al., 2007). Carotenoids were considered to be free radical scavengers, which can reduce the damage caused by stress because it can not only participate in the plant hormones biosynthesis process, but also participate in the defense of chemical synthesis (Rolland et al., 2012). The direct substrate for the synthesis of plant hormone abscisic acid (ABA) in the carotenoid biosynthesis pathway and violaxanthin de-epoxidase is 9-cis-epoxy carotenoid dioxygenase, which is a key component of the lutein cycle (Lu and Li, 2008). In our study, both the key enzymes 9-cis-epoxycarotenoid dioxygenase (Ma06_g22020, EC:1.13.11.51) and violaxanthin de-epoxidase (Ma04_g19490, EC:1.23.5.1) in the carotenoid biosynthesis pathway were found to be up-regulated. Therefore, we believe that the changes of carotenoid content and composition will lead to direct changes in plant physiological and biochemical processes.

Plants can produce reactive oxygen species (ROS). ROS can damage membrane structure, destroy biological macromolecules, and lead to metabolic disorders. In serious cases, it will cause plants to die under pressure. Superoxide dismutase (SOD) and peroxidase (POD) are antioxidant enzymes in plants. Their major function is to eliminate ROS free radicals, hold back ROS immoderate accumulation, prevent or reduce the attack of ROS on the membrane and alleviate damage to the membrane (Khan et al., 2017). In the KEGG enrichment pathway sequences, we found some DEGs that code for SOD and POD, and which can lead to physiological and biochemical process changes in salt-stress BD banana plant.

In terms of mechanical or environmental damage (such as drought, ultraviolet, or injury), phenylpropane- and phenylpropane-based secondary products (such as lignin, hematoxylin, flavonoids, or condensed tannins) accumulate in the plant (Liu et al., 2015). Here, we found that all transcripts encoding key enzymes in the phenylpropane biosynthesis pathway were found to be significantly up-regulated and were composed of four phenylalanine ammonia lyase (PAL) family genes: *omega-hydroxyPALmitate O-feruloyl transferase-like* (Ma10_g20660), *phenylalanine ammonia-lyase-like* (Ma01_g04420, Ma11_g14940), *fatty acid desaturase 4, chloroplastic-like* (Ma10_g04770); one *isoforms of 4-coumarat*e*: CoA ligase* (4CL): *4-coumarate–CoA ligase-like 3* (Ma11_g13120); *chalcone synthase* (CHS): *chalcone synthase 2-like* (Ma06_g09780), and *flavonol synthase* (FLS): *flavonol synthase/flavanone 3-hydroxylase* (Ma03_g06970), indicating these biosynthesis pathway genes may play a significant function in BD banana facing salt-stress response.

Transcription factors are involved in responses induced by biotic and abiotic stress tolerance of plants, and they regulate gene expression as an upstream regulator in the metabolic pathways (Shan et al., 2013). Here, we clarified that some transcription factor family member genes consist of MYB, NAC, bHLH, WRKY, etc. These genes have been functionally annotated in the response of BD banana to salt stress. Transgenic plants with overexpression of these TFs can enhance the stress resistance of these transgenic plants (Hao et al., 2011; Shen et al., 2012; Sakuraba et al., 2015; Wang et al., 2018). It was reported that many ERF, ZFP, and WRKY genes from various species play an active function in plants facing biotic or abiotic stress environments. IbZFP1 has been reported to participate in salt stress and drought-stress tolerance in transgenic *Arabidopsis*, and overexpression of IbZFP1gene could improve the tolerance of salt stress and drought stress in *Arabidopsis* (Wang et al., 2016). WRKY transcription factors will definitely participate in plant’s transcriptional reprogramming when facing the abiotic or biological stress environment (Rushton et al., 2010). It was reported that the overexpression of *MusaWRKY71* in banana plants could enhance the tolerance of transgenic banana to both oxidation stress and salt stress (Shekhawat and Ganapathi, 2013). This evidence revealed the crucial and highly efficient transcriptional regulation mechanism adjusted by TFs, which helps them enhance their tolerance in biotic or abiotic stress environment. MYB and bHLH transcription factors belong to large TFs groups that can activate CHS and FLS promoters. Both bZIP and MYB transcription factors can enhance stress response also (Sablowski et al., 1994; Mehrtens et al., 2005; Liu et al., 2015). The accumulation of these TFs might promote salt stress tolerance of BD banana. Molecular analysis showed that ABA and JA, AUX, cytokinin, BR, and SA regulated the expression of many genes under osmotic stress (Zhang et al., 2006). Udvardi hypothesized that the rise of hormone levels would activate the different DEGs and then adjust the down-stream transcription factors, including MYB, WRKY, bHLH, AP2/ER-EBP, C2H2, etc., thereby regulating ion transports, stimulating stomatal closure, and increasing water absorption (Udvardi et al., 2007). All these confirmed that TFs play a key function in banana salt stress tolerance.

## Conclusion

The salinization of soil is a widespread environmental problem. Banana (*Musa acuminata* L.) is a salt-sensitive plant whose growth, development, and production are constrained by salt stresses. This study analyzed the phenotypic, physiological, and transcriptome changes of a main agriculture banana variety (*Musa acuminata* cv. BD) with 60 mM NaCl stress treatment. The purpose of this study was to clarify the influence of a physiological change of BD banana induced by salt stress at the transcriptome level. The fluctuation of root activity, MDA, Pro content, and antioxidant enzymes activity in salt treatment BD banana plant indicate that there was a material change in salt-sensitive gene expression that will happen at 10 days after treatment with 60 mM NaCl. Therefore, RNA-seq analysis was performed on these leaf samples (i.e., 0 and 10 days). Many DEGs were identified under salinity stress in leaf tissue. In all 3,378 DEGs were identified in the leaves of BD banana plant. KEGG analysis showed that these DEGs genes relating to phenylpropanoid biosynthesis, ribosome, starch and sucrose metabolism, amino sugar, and plant hormone signal transduction had simultaneously changed the expression under salt stress. These genes may be related to promoting the growth of BD banana against salt stress atmosphere. DEGs in the process of phenylpropanoid biosynthesis, starch and sucrose metabolism, and amino sugar and plant hormone signal transduction are specifically regulated in response to salt stress response. Totally, 48 differentially expressed TFs, including WRKY, MYB, NAC, and bHLH, were annotated in salt stress conditions. Here, we found that all transcripts encoding key enzymes in the phenylpropane biosynthesis pathway were found to be significantly up-regulated, indicating that the genes in these pathways may play a significant function in BD banana faced to the salt stress response. The results of this study laid a foundation for developing agriculturally main banana varieties with salt tolerance by using genetic engineering technology and an effective breeding scheme. The study on the genetic expression pattern of BD banana under salt stress is helpful to understand the regulatory network of salt stress adaptation of banana plants, and to choose appropriate candidate genes for operation to improve the salt tolerance of banana plants.

## Data Availability Statement

The data presented in the study are deposited in the NCBI repository, accession number PRJNA579479.

## Author Contributions

JW and DL designed the research and wrote and revised the manuscript. JW and YL prepared the research materials and performed the experiments. SW and DL provided funds and experimental conditions. All authors read and approved the final manuscript.

## Conflict of Interest

The authors declare that the research was conducted in the absence of any commercial or financial relationships that could be construed as a potential conflict of interest.

## Publisher’s Note

All claims expressed in this article are solely those of the authors and do not necessarily represent those of their affiliated organizations, or those of the publisher, the editors and the reviewers. Any product that may be evaluated in this article, or claim that may be made by its manufacturer, is not guaranteed or endorsed by the publisher.
